# Enhanced antimicrobial de-escalation for pneumonia in mechanically ventilated patients: a cross-over study

**DOI:** 10.1186/s13054-017-1772-4

**Published:** 2017-07-15

**Authors:** Tracy Trupka, Kristen Fisher, Scott T. Micek, Paul Juang, Marin H. Kollef

**Affiliations:** 1 Division of Pulmonary and Critical Care Medicine, Washington University School of Medicine, 4523 Clayton Ave, Campus Box 8052, St. Louis, MO 63110 USA; 2 Division of Pharmacy Practice, St. Louis College of Pharmacy, St. Louis, MO USA

**Keywords:** Pneumonia, Mechanical ventilation, Antibiotics, De-escalation

## Abstract

**Background:**

Antibiotics are commonly administered to hospitalized patients with infiltrates for possible bacterial pneumonia, often leading to unnecessary treatment and increasing the risk for resistance emergence. Therefore, we performed a study to determine if an enhanced antibiotic de-escalation practice could improve antibiotic utilization in mechanically ventilated patients with suspected pneumonia cared for in an academic closed intensive care unit (ICU).

**Methods:**

This was a prospective cross-over trial comparing routine antibiotic management (RAM) and enhanced antimicrobial de-escalation (EAD) performed within two medical ICUs (total 34 beds) at Barnes-Jewish Hospital, an academic referral center. Patients in the EAD group had their antibiotic orders and microbiology results reviewed daily by a dedicated team comprised of a second-year critical care fellow, an ICU attending physician and an ICU pharmacist. Antibiotic de-escalation recommendations were made when appropriate based on microbiologic test results and clinical response to therapy.

**Results:**

There were 283 patients evaluable, with suspected pneumonia requiring mechanical ventilation: 139 (49.1%) patients in the RAM group and 144 (50.9%) in the EAD group. Early treatment failure based on clinical deterioration occurred in 33 (23.7%) and 40 (27.8%) patients, respectively (*P* = 0.438). In the remaining patients, antimicrobial de-escalation occurred in 70 (66.0%) and 70 (67.3%), respectively (*P* = 0.845). There was no difference between groups in total antibiotic days ((median (interquartile range)) 7.0 days (4.0, 9.0) versus 7.0 days (4.0, 8.8) (*P* = 0.616)); hospital mortality (25.2% versus 35.4% (*P* = 0.061)); or hospital duration (12.0 days (6.0, 20.0) versus 11.0 days (6.0, 22.0) (*P* = 0.918).

**Conclusions:**

The addition of an EAD program to a high-intensity daytime staffing model already practicing a high-level of antibiotic stewardship in an academic ICU was not associated with greater antibiotic de-escalation or a reduction in the overall duration of antibiotic therapy.

**Trial registration:**

ClinicalTrials.gov, NCT02685930. Registered on 26 January 2016.

**Electronic supplementary material:**

The online version of this article (doi:10.1186/s13054-017-1772-4) contains supplementary material, which is available to authorized users.

## Background

Escalating rates of antibiotic resistance add substantially to the morbidity, mortality, and costs related to infection in hospitalized patients, especially those in the intensive care unit (ICU) setting [1–5]. The rapid evolution of antibiotic resistance impedes efforts to insure that initial appropriate antibiotic therapy (IAAT) is delivered to critically ill infected patients. IAAT is a key determinant of outcome in severe infection and the Surviving Sepsis Guidelines strongly support initiatives to promote and facilitate timely appropriate antibiotic treatment to reduce mortality [6, 7]. The rising rates of antibiotic resistance have likely contributed to the increase in mortality attributed to antibiotic-resistant bacteria despite the overall reduction in deaths attributed to infectious diseases in the last century [8].

Because not all serious infections are due to multidrug-resistant (MDR) organisms, clinicians must have a strategy for determining which patients should receive empiric broad-spectrum antibiotics [9]. The practice of antibiotic de-escalation has emerged as an antibiotic decision-making strategy in the ICU balancing the need for IAAT, in order to improve patient outcomes, with the need for avoidance of unnecessary antibiotics so as to reduce resistance emergence [10]. De-escalation generally refers to tailoring of empirical antibiotic treatment according to the susceptibilities of the bacteria isolated, selecting the narrowest spectrum antibiotic or stopping antibiotics altogether if non-infectious etiology is established.

Given the importance of balancing IAAT with the avoidance of unnecessary antibiotic exposure, we performed a clinical trial with the goal of determining whether an enhanced antimicrobial de-escalation (EAD) practice could improve antibiotic utilization and outcomes in mechanically ventilated patients with suspected pneumonia. Our hypothesis was that introduction of an EAD practice would increase rates of antimicrobial de-escalation in the ICU population. We also wanted to determine whether a practice of EAD impacted other outcomes including mortality and lengths of stay.

## Methods

### Study population and data source

The study was conducted within the two medical ICUs (total 34 beds). at Barnes-Jewish Hospital, an academic referral center of 1250 beds. The medical ICUs are geographically co-located closed units with shared physician, nursing, pharmacist, and respiratory therapist staff. The medical ICUs are staffed 24 hours per day and 7 days per week by these teams, including intensivists board-certified in critical care. This investigation was approved by the Washington University School of Medicine Human Studies Committee and the need for informed consent was waived (Institutional Review Board (IRB)# 201509075; ClinicalTrials.gov Identifier: NCT02685930). All mechanically ventilated patients with suspected pneumonia, from 1 January 2016 through 31 December 2016 were eligible for inclusion. Patients were excluded if they had a concomitant non-pulmonary infection requiring antibiotic therapy, bronchiectasis, or significant immune suppression. Data were prospectively collected from the electronic health record and from patients’ ICU teams.

### Study outcomes/objectives

The primary objective of this study was to determine whether and how frequently antibiotic de-escalation occurred following the initial prescription of antibiotics for suspected pneumonia. The secondary outcomes assessed included antibiotic duration, failure of initial antibiotic therapy, hospital mortality, ICU and hospital duration, and subsequent infection with antibiotic-resistant strains of bacteria.

### Definitions and study design

The two medical ICUs were initially randomly assigned so that subjects admitted to each ICU would receive either routine antibiotic management (RAM) for suspected pneumonia or EAD. Patients in the RAM group had all antibiotic decisions determined by their ICU team composed of a critical care board-certified attending physician, a critical care fellow, resident physicians, and a critical care specialty pharmacist. Antibiotic decision-making was similar in patients in the EAD group except that all antibiotic orders and microbiology results were reviewed on a daily basis by the study team (TT, KF, STM, PJ, and MHK) to specifically determine if further antibiotic de-escalation could occur. The study team performed these reviews from 7 a.m. to 6 p.m. on weekdays excluding weekends and holidays. After 6 months the two medical ICUs were crossed over in terms of the type of antibiotic management strategy delivered to patients.

Adult patients (age >18 years) were identified prospectively with suspected pneumonia (community-onset pneumonia (COP), hospital-acquired pneumonia (HAP), or ventilator-associated pneumonia (VAP)) in accordance with the American Thoracic Society position statement on pneumonia [11]. The diagnosis included presence of a new or progressive radiographic infiltrate and at least two of the following clinical features: fever >38 °C, leukocytosis (>10 × 10^9^cells/L), leukopenia (≤4 × 10^9^cells/L), or purulent secretions. The presence of a new or progressive radiographic infiltrate was based on the interpretation of the chest radiograph by board-certified radiologists blinded to the study. Patients admitted from the community without prior radiographs were assumed to have a new infiltrate when present. Pneumonia was classified microbiologically according to the pathogen(s) identified as pathogen-negative, viral, antibiotic-susceptible, or antibiotic-resistant. For purposes of this investigation, antibiotic-susceptible was determined according to ceftriaxone susceptibility, as ceftriaxone represents the antimicrobial agent most frequently recommended for hospitalized patients with pneumonia coming from the community setting [12]. Additionally, patients could be classified as having non-infectious illness mimicking suspected pneumonia (see Additional file 1: Table S1). Patients classified as having viral pneumonia had to meet the aforementioned clinical and radiographic criteria for pneumonia.

Early failure of initial antibiotic therapy was defined as progression to shock, mortality, or progression of the radiographic infiltrates without resolution of clinical parameters (leukocytosis or leukopenia, fever, purulent secretions) within 48 hours of starting antibiotic therapy. Septic shock was defined as the need for vasopressors (norepinephrine, dopamine, vasopressin, epinephrine, or phenylephrine). Only the first episode of suspected pneumonia was recorded. Antimicrobial treatment was classified as IAAT if the initial regimen had in vitro activity demonstrated against the isolated pathogens. Immune suppression was defined as acquired immune deficiency syndrome, solid organ or bone marrow transplant, hematologic malignancies, solid cell cancers treated with chemotherapy or radiation, long-term corticosteroids (>10 mg/day), and other immunosuppressive drugs (e.g., biologics for rheumatologic disorders). Multidrug-resistant (MDR) pathogens had to demonstrate in vitro resistance to at least one agent from three distinct classes of antimicrobials that would normally have activity against that bacterium [13].

Antimicrobial de-escalation was defined as a change in the initially prescribed antibiotic regimen resulting in one of the following: (1) a reduction in the number of antibiotics prescribed, (2) elimination of antibiotic coverage for a class of pathogens (e.g., stopping coverage for *Staphylococcus aureus*), and (3) changing the antibiotic regimen to a more narrow-spectrum regimen. For purposes of this investigation, antibiotics were ranked according to their activity spectrum against pneumonia pathogens, emphasizing Gram-negative bacteria (5, highest; 0, lowest) and Gram-positive bacteria (1, highest; 0, lowest) (Table 1). For combination regimens, rank was assigned according to the most potent drug. Therapy escalation was defined as the switch to or addition of a drug class or classes with a broader spectrum (using definitions in Table 1).

**Table 1:** Antibiotic ranking according to activity spectrum against Gram-negative and Gram-positive bacteria associated with pneumonia

Gram-negative antibiotic	Rank	Gram-positive antibiotic	Rank
Carbapenem	5	Vancomycin, linezolid, ceftaroline	1
Cefepime	4		
Ureidopenicillin/monobactam	3		
Quinolone (ciprofloxacin or levofloxacin)	2		
Ceftriaxone	1		
None	0	None	0

### Antimicrobial monitoring

From January 2002 through the present, Barnes-Jewish Hospital utilized an antibiotic control program to help guide antimicrobial therapy for bacterial infections. During this time, the use of cefepime, gentamicin, or vancomycin was unrestricted. However, initiation of intravenous ciprofloxacin, imipenem, meropenem, piperacillin/tazobactam, ceftolozone/tazobactam, ceftazidime/avibactam, linezolid, ceftaroline, or daptomycin was restricted and required preauthorization from either a clinical pharmacist or infectious diseases physician. Each intensive care unit (ICU) had a clinical pharmacist who reviewed all antibiotic orders to ensure that dosing and interval of antibiotic administration was adequate for individual patients based on body size, renal function, and the resuscitation status of the patient. The duration of antibiotic therapy was at the discretion of the ICU treating team based on the patient’s microbiology results and response to therapy.

The initial antibiotic dosages employed for the treatment of bacterial infections at Barnes-Jewish Hospital were as follows: cefepime, 1 to 2 g every 8 hours; piperacillin–tazobactam, 4.5 g every 6 hours; imipenem, 0.5 g every 6 hours; meropenem, 1 to 2 g every 8 hours; ceftolozone/tazobactam, 1.5 or 3 g every 8 hours; ceftazidime/avibactam, 2.5 g every 8 hours; ciprofloxacin, 400 mg every 8 hours; levofloxacin, 750 mg once daily; vancomycin, 15 mg/kg every 12 hours; linezolid, 600 mg every 12 hours; and ceftaroline, 600 mg every 8 hours. Subsequent antibiotic dose and frequency were adjusted, where appropriate.

### Antimicrobial susceptibility testing

The microbiology laboratory performed antimicrobial susceptibility of the bacterial isolates using the disk diffusion method according to guidelines and breakpoints established by the Clinical Laboratory and Standards Institute and published during the inclusive years of the study [14]. All classifications of antibiotic resistance were based on in vitro susceptibility testing using these established breakpoints. Viral identification was obtained using the Biofire FilmArray® Respiratory Panel (bioMérieux, Marcy l'Etoile, France).

### Statistical analyses

The sample size was determined by a convenience sample of all mechanically ventilated patients with suspected pneumonia identified in the medical ICUs during the study period. Continuous variables were expressed as mean and standard deviation (SD) or median and interquartile range (IQR) when appropriate. The *t* test was used to analyze normally distributed continuous variables, whereas the Mann–Whitney *U* test was used to analyze non-normally distributed continuous variables. Categorical data were reported as frequency distributions and analyzed using the chi-square test. *P* values less than 0.05 were considered statistically significant, and all tests were two-tailed. All analyses were done using SPSS Statistics 21 (IBM SPSS Statistics, Version 21.0. Armonk, NY, USA).

## Results

**Fig. 1:**
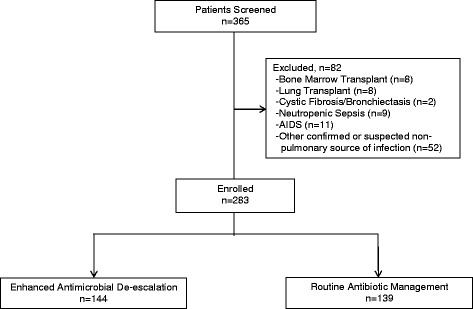
Consolidated Standards of Reporting Trials (CONSORT) flow diagram. There were seven patients with more than one exclusion criterion

Three hundred sixty-five consecutive patients with respiratory failure and pneumonia were identified. The main reasons for ICU admission in these patients included COP (*n* = 237; 64.9%), chronic obstructive pulmonary disease (*n* = 46; 12.6%), congestive heart failure (*n* = 31; 8.5%), renal failure (*n* = 19; 5.2%), and altered mental status (*n* = 15; 4.1%). HAP occurred in 99 patients (27.1%) and VAP in 29 patients (7.9%). Of the 365 screened patients, 82 (22.5%) were excluded due to the presence of immune suppression, bronchiectasis, or the presence of another non-pulmonary infection requiring antibiotic therapy (Fig. 1).

The final study population comprised 283 patients of whom 144 (50.9%) were in the EAD group and 139 (49.1%) in the RAM group. The majority were white men admitted from home or transferred from an outside hospital, with approximately 10% of the patients residing in a nursing home or rehabilitation facility before admission (Table 2). There were 100 patients (35.3%) classified as having pathogen-negative pneumonia, 49 (17.3%) as having viral pneumonia, 53 (18.7%) as having antibiotic-resistant pneumonia, 40 (14.1%) as having antibiotic-susceptible pneumonia, and 41 (14.5%) as having illness of non-infectious etiology. The distribution of pneumonia categories was similar in both study groups (Table 3). The pathogens identified are shown in Additional file 1: Table S2. *S. aureus* (17.0%) was the most common pathogen associated with pneumonia followed by rhinovirus (12.4%), *Pseudomonas aeruginosa* (9.8%), and influenza A (6.2%).

**Table 2:** Baseline characteristics of the study population

	Enhanced antibiotic de-escalation (*n* = 144)	Routine antibiotic management (*n* = 139)	*P* value
Age	61 (52, 70)	60 ([45, 71)	0.494
Gender			
Male	79 (54.9)	80 (57.6)	0.648
Female	65 (45.1)	59 (42.4)	
Race			
Caucasian	87 (60.4)	82 (59.0)	0.523
African American	50 (34.7)	54 (38.8)	
Other	7 (4.0)	3 (2.1)	
APACHE II score	22.7 ± 7.3	21.7 ± 7.8	0.262
Charlson Comorbidity Index	2.0 (1.0, 4.8)	2.0 (1.0, 5.0)	0.744
Clinical Pulmonary Infection Score	7.2 ± 2.6	7.3 ± 2.4	0.730
Congestive heart failure	22 (15.3)	10 (14)	0.701
Chronic obstructive lung disease	42 (29.2)	41 (29.5)	0.951
Interstitial lung disease	6 (4.2)	5 (3.6)	0.804
Cirrhosis	8 (5.6)	6 (4.3)	0.631
Diabetes	41 (28.5)	48 (34.5)	0.272
End-stage renal disease	11 (7.6)	7 (5.0)	0.370
Malignancy	35 (24.3)	39 (28.1)	0.473
HIV	2 (1.4)	4 (2.9)	0.385
Solid organ transplant	5 (3.5)	4 (2.9)	0.776
Home steroids	20 (13.9)	27 (19.4)	0.211
Other home immunosuppressant	16 (11.1)	15 (10.8)	0.931
Admission source			
Home	58 (40.3)	46 (33.1)	0.213
Transfer from another hospital	38 (26.3)	38 (27.3)	
Transfer from lower level of care	30 (20.8)	36 (25.9)	
Nursing home/skilled nursing	10 (6.9)	12 (8.6)	
Lateral ICU transfer	2 (1.4)	6 (4.3)	
Long-term acute care hospital	2 (1.4)	1 (0.7)	
Inpatient rehabilitation facility	4 (2.8)	0 (0.0)	
Prior hospitalization			
Within 6 months	74 (51.4)	69 (49.6)	0.769
Never or not within 6 months	70 (48.6)	70 (50.4)	
Prior antibiotic exposure			
Within prior 30 days	76 (52.8)	64 (46.0)	0.257
Not within prior 30 days	68 (47.2)	75 (54.0)	

Values expressed as median (interquartile range), mean ± standard deviation, or number (percent). *APACHE* Acute Physiology and Chronic Health Evaluation, *HIV* human immune deficiency virus, *ICU* intensive care unit

**Table 3:** Pneumonia classification

	Enhanced antibiotic de-escalation (*n* = 144)	Routine antibiotic management (*n* = 139)	*P* value
Pathogen-negative	51 (35.4)	49 (35.3)	0.592
Viral	21 (14.6)	28 (20.1)	
Ceftriaxone-resistant	31 (21.5)	22 (15.8)	
Ceftriaxone-sensitive	19 (13.2)	21 (15.1)	
Non-infectious etiology	22 (15.3)	19 (13.7)	

Values expressed as number (percent)

Antibiotic de-escalation occurred in 140 patients (49.5%) including a reduction in the number of antibiotics prescribed or elimination of antibiotic coverage for a class of pathogens in 39 patients (27.9%) and changing the antibiotic regimen to a narrower-spectrum regimen in 101 patients (72.1%) (rank 5 or 4 Gram-negative antibiotic changed to a rank 3, 2 or 1 Gram-negative antibiotic as defined in Table 1 in all 101 patients). Antibiotic de-escalation occurred statistically more often in patients without septic shock compared to those with septic shock (61.6% versus 44.6%; *P* = 0.001). There was no statistical difference in antibiotic de-escalation according to double empiric coverage for Gram-negative bacteria (*n* = 10) versus single-agent coverage (*n* = 273) (20.2% versus 50.5%; *P* = 0.103). Antibiotic escalation took place in 16 patients (5.6%) due to the identification of a pathogen resistant to the initial antibiotic regimen, and no change in antibiotic therapy occurred in 127 (44.9%) patients. Hospital mortality was greatest among patients undergoing antibiotic escalation (50.0%) followed by patients having no change in antibiotic therapy (43.3%), and was lowest for patients receiving antibiotic de-escalation (16.4%) (*P* < 0.001).

**Fig. 2:**
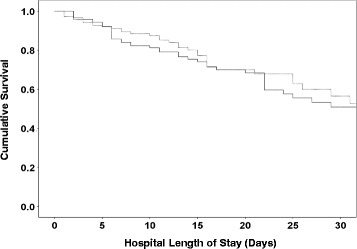
Kaplan–Meier curves for cumulative survival in the routine antibiotic management group (*hatched line*) and the enhanced antimicrobial de-escalation group (*solid line*) (log-rank test = 0.122)

Early failure of antibiotic therapy occurred in 73 patients (25.8%) and was similar between study groups (Table 4). Among the remaining 210 patients eligible clinically for antimicrobial de-escalation, antibiotic de-escalation was similar for patients in the EAD and RAM groups. There was no difference between study groups in total antibiotic days, hospital mortality, or hospital duration. The occurrence of secondary pneumonia and secondary pneumonia attributed to ceftriaxone-resistant bacteria was also similar in both groups (Table 4). Kaplan–Meier curves comparing survival showed no difference in mortality in the RAM group compared to the EAD group (Fig. 2).

**Table 4:** Antibiotic and clinical outcomes

	Enhanced antibiotic de-escalation (*n* = 144)	Routine antibiotic management (*n* = 139)	*P* value
Early failure of initial antibiotics	40 (27.8)	33 (23.7)	0.438
Antibiotics de-escalated*	70/104 (67.3)	70/106 (66.0)	0.845
Deterioration post de-escalation*	8/70 (11.4)	6/70 (8.6)	0.573
Total antibiotic days	7.0 (4.0, 8.8)	7.0 (4.0, 9.0)	0.616
Mortality	51 (35.4)	35 (25.2)	0.061
ICU length of stay	6.0 (3.0, 12.0)	6.0 (4.0, 12.0)	0.935
Hospital length of stay	11.0 (6.0, 22.0)	12.0 (6.0, 20)	0.918
Ventilator days	4.5 (2.0, 9.0)	4.0 (2.0, 9.0)	0.953
Secondary pneumonia	12 (8.3)	12 (8.6)	0.928
Secondary pneumonia due to antibiotic-resistant pathogen	9 (6.3)	6 (4.3)	0.468

Values expressed as median (interquartile range) and number (percent). *ICU* intensive care unit. *The percentages were derived from the subgroups of patients who did not have failure of initial antibiotics

## Discussion

We found that the addition of an EAD program to a high-intensity ICU staffing model already practicing antimicrobial de-escalation was not associated with greater antibiotic de-escalation or a reduction in the overall duration of antibiotic therapy in patients with suspected pneumonia during mechanical ventilation. We also observed no significant effect on hospital mortality, lengths of stay, or subsequent occurrence of antibiotic-resistant infections.

We selected antibiotic de-escalation as the primary outcome for our investigation of an EAD program. However, as emphasized in several recent systematic reviews, antibiotic de-escalation is dependent on multiple factors, such as risk for infection with resistant pathogens, severity of illness, clinical response to initial therapy, site of infection, microorganisms associated with infection, adequacy of source control, and institutional practice patterns [10, 15]. Moreover, antibiotic de-escalation is accepted as a part of the global antimicrobial stewardship approach, inclusive of other elements such as the route and mode of antibiotic administration and the total duration of antimicrobial therapy [15]. The problem for intensivists is that delaying the administration of IAAT is associated with greater mortality [6]. Therefore, as recommended by the Surviving Sepsis Campaign, likely pathogens associated with sepsis should initially be covered for with the empiric antibiotic regimen until microbiologic results become available [7]. This creates a situation that often promotes the empiric administration of broad-spectrum antibiotics. The advent of rapid diagnostics is likely to facilitate the implementation of EAD in the future with the more timely availability of pathogen identification and antibiotic susceptibility compared to current approaches [10, 16].

One major area of continuing uncertainty for ICUs implementing antimicrobial stewardship programs is the concept of antimicrobial de-escalation. Further research must be done to determine what defines antimicrobial de-escalation, when it is appropriate, how to integrate infectious disease consultation into the ICU, when to “re-escalate” therapy, and how to transition antimicrobial decision-making when patients move from the ICU to the hospital ward [10, 17]. At present there are no established guidelines or benchmarks for de-escalation practices in critically ill patients that could serve as a reference point for ICU clinicians. Antimicrobial de-escalation is also uncertain in pathogen-negative sepsis, particularly whether it is safe to de-escalate and the optimal duration of antimicrobials. With improving culture methods and rapid diagnostics the proportion of patients with pathogen-negative sepsis will also likely diminish over time, resulting in fewer such uncertainties [18]. We chose to include patients with viral pneumonia in our study given that viral causes of pneumonia are increasingly recognized with the advent of new rapid diagnostics and they represent an opportunity for de-escalating traditional antibiotic therapy.

Several limitations of our study should be recognized. First, the study design did not allow the research team to supplant the ICU teams in terms of overall antibiotic decision-making. The study team could only make recommendations to the ICU teams on de-escalation. Furthermore, the EAD study team was only available during daytime hours and not during weekends or holidays. This may have had an impact on the study team’s ability to influence overall antibiotic management in patients with pneumonia during the time periods the study team was not available. Second, the EAD team and the ICU teams comprised similar clinicians including intensivists and pharmacists with similar levels of training and experience. This may have contributed to our inability to detect any impact of the EAD program on antibiotic management or clinical outcomes. Based on prior experience, inclusion of an infectious disease specialist or microbiologist might have improved our ability to improve antibiotic utilization with the EAD program [19, 20]. Third, we had a relatively small number of patients with VAP compared to COP and HAP. However, most of the patients with COP in our study had at least one healthcare-associated risk factor (prior hospitalization, admission from a nursing facility, immune suppression, or prior antibiotic exposure) placing them at higher risk for infection with potentially antibiotic-resistant bacteria.

Another limitation of our study is that the data were derived from a single center, and this necessarily limited the generalizability of our findings. As such, our results may not reflect what one might see at other institutions. For example, all of the ICUs at Barnes-Jewish Hospital are closed units with high-intensity multidisciplinary staffing providing continuous patient care. There is also a long history of antimicrobial stewardship being practiced within these ICUs [21–23]. The organizational makeup of the ICUs may also have contributed to the negative findings of our study. The presence of high-intensity staffing models in ICUs has previously been suggested as a possible explanation for other negative trials performed in critically ill patients [24, 25]. Moreover, our study was performed in two medical ICUs. It is possible that our findings might have differed had we selected other types of ICUs, due to differences in case mix, severity of illness, and practice patterns. Last, we did not directly integrate the use of a biomarker such as procalcitonin into the EAD program, which might have allowed further antimicrobial de-escalation and/or reductions in antibiotic duration.

## Conclusions

In conclusion, we found that the addition of an EAD program to a high-intensity staffing model in medical ICUs was not associated with greater antibiotic de-escalation or a reduction in the overall duration of antibiotic therapy in mechanically ventilated patients with suspected pneumonia. Future studies are needed to determine whether EAD programs can impact antibiotic management in other types of ICUs which may have a lesser focus on de-escalation practices and ICUs with different staffing models. Additionally, identifying the optimal methods for carrying out antimicrobial de-escalation in the ICU setting, to include the integration of infectious disease experts and rapid diagnostics, deserves further investigation. Until such studies are performed, critical care clinicians should incorporate antimicrobial review strategies into their daily practice in order to balance the need for administering IAAT and the avoidance of unnecessary antibiotic utilization.

## Additional file

Additional file 1: Table S1. Non-infectious etiologies in patients with suspected pneumonia. **Table S2**. Respiratory microbiology. (DOCX 26 kb)
