# Supplementary material for: Enhanced antimicrobial de-escalation for pneumonia in mechanically ventilated patients: a cross-over study
Source: Crit Care. 2017 Jul 15;21:180. doi: 10.1186/s13054-017-1772-4 (PMC5513164; doi:10.1186/s13054-017-1772-4)
Supplement: Additional file 1: Table S1. — Non-infectious etiologies in patients with suspected pneumonia. Table S2. Respiratory microbiology. (DOCX 26 kb) [file 13054_2017_1772_MOESM1_ESM.docx]

Additional file 1: Table S1. Non-Infectious Etiologies for Patients With Suspected Pneumonia

|  |  |
| --- | --- |
| Atelectasis | Resolution of radiographic and clinical signs of suspected pneumonia within 48 hours with lung recruitment maneuvers and the absence of microbiologically confirmed pathogens consistent with pneumonia. |
| Hydrostatic pulmonary edema (cardiogenic or fluid overload due to renal failure) | Resolution of the signs and symptoms of suspected pneumonia within 48 hours with the administration of diuretics or renal replacement therapy and the absence of microbiologically confirmed pathogens consistent with pneumonia. |
| Acute respiratory distress syndrome | Pulmonary edema meeting the Berlin definition and the absence of microbiologically confirmed pathogens consistent with pneumonia (1). |
| Aspiration | Presence of a witnessed aspiration event and the absence of microbiologically confirmed pathogens consistent with pneumonia. |
| Pulmonary embolism with infarction | Radiographic confirmation of pulmonary embolism with the absence of microbiologically confirmed pathogens consistent with pneumonia. |
| Chronic obstructive pulmonary disease | Resolution of radiographic and clinical signs of suspected pneumonia within 48 hours with the administration of bronchodilators and corticosteroids. |

1. ARDS Definition Task Force, Ranieri VM, Rubenfeld GD, et al: Acute respiratory distress syndrome: the Berlin Definition. *JAMA* 2012; 307:2526-2533.

Additional file 1: Table S2. Respiratory Microbiology

| Enhanced Antibiotic De-escalation  (n=144) | | Routine Antibiotic Management  (n=139) | |
| --- | --- | --- | --- |
| Nonviral Pathogen |  |  |  |
| *Staphylococcus aureus* | 17 | *Staphylococcus aureus* | 16 |
| *Pseudomonas aeruginosa* | 12 | *Pseudomonas aeruginosa* | 7 |
| *Streptococcus pneumoniae* | 6 | *Streptococcus* spp. – other | 7 |
| *Escherichia coli* | 4 | *Streptococcus pneumoniae* | 3 |
| *Stenotrophomonas maltophilia* | 3 | *Haemophilus influenzae* | 3 |
| *Klebsiella pneumoniae* | 3 | *Stenotrophomonas maltophilia* | 3 |
| *Legionella pneumophila* | 3 | *Enterobacter* spp. | 3 |
| *Aspergillus* species | 3 | *Klebsiella pneumoniae* | 2 |
| *Enterobacter* spp. | 2 | *Moraxella catarrhalis* | 2 |
| *Moraxella catarrhalis* | 2 | *Escherichia coli* | 1 |
| *Providencia stuartii* | 2 | *Enterococcus faecium* | 1 |
| *Pneumocystis jiroveci* | 2 | *Acinetobacter* species | 1 |
| *Serratia marcescens* | 1 | *Chryseobacterium* spp | 1 |
| *Haemophilus influenzae* | 1 | *Providencia rettgeri* | 1 |
| *Proteus mirabilis* | 1 | *Citrobacter koseri* | 1 |
| *Streptomyces* spp. | 1 | *Aspergillus* species | 1 |
| *Streptococcus* spp.– other | 1 | *Blastomyces dermatididis* | 1 |
|  |  | *Histoplasmosis capsulatum* | 1 |
| Viral Pathogen |  |  |  |
| Rhinovirus/Enterovirus | 8 | Rhinovirus/Enterovirus | 16 |
| Coronavirus | 5 | Influenza A | 10 |
| Respiratory Syncytial Virus | 4 | Coronavirus | 4 |
| Herpes Simplex Virus | 4 | Respiratory Syncytial Virus | 3 |
| Adenovirus | 3 | Metapneumovirus | 3 |
| Influenza A | 2 | Parainfluenza | 3 |
| Metapneumovirus | 2 | Herpes Simplex Virus | 3 |
| Cytomegalovirus | 2 | Adenovirus | 1 |
| Influenza B | 1 | Cytomegalovirus | 1 |
| Multiple pathogens | 20 | Multiple pathogens | 23 |

Among the enhanced de-escalation group 70 had positive cultures for respiratory pathogens compared to 72 in the routine antibiotic management group.
